# Dermoscopy Clues in Pigmented Bowen's Disease

**DOI:** 10.1155/2010/464821

**Published:** 2010-09-16

**Authors:** Daniela Gutiérrez-Mendoza, Roberto Narro-Llorente, Marcia Karam-Orantes, Verónica Fonte-Avalos, Eduwiges Martínez-Luna, Sonia Toussaint-Caire, Judith Domíguez-Cherit

**Affiliations:** ^1^Department of Surgical Dermatology, Hospital General “Dr. Manuel Gea González”, Calzada de Tlalpan 4800, Col. Toriello Guerra, Tlalpan, CP 14000, D.F., México, Mexico; ^2^Department of Dermatology, Hospital General “Dr. Manuel Gea González”, Calzada de Tlalpan 4800, Col. Toriello Guerra, Tlalpan, CP 14000, D.F., México, Mexico; ^3^Department of Dermatopathology, Hospital General “Dr. Manuel Gea González”, Calzada de Tlalpan 4800, Col. Toriello Guerra, Tlalpan, CP 14000, D.F., México, Mexico

## Abstract

Pigmented tumors have similar clinical features that overlap and hamper diagnosis. Dermoscopy increases the diagnostic accuracy of doubtful melanocytic lesions and has been used as a noninvasive tool in the detection of pigmented lesions (PLs) like melanoma, basal cell carcinoma, and pigmented Bowen's disease (pBD). Our objective was to show the dermoscopic features of 2 cases of pBD and compare with the findings reported in the literature. Two dermoscopic images of biopsy proven pBD were retrospectively analyzed for dermoscopic patterns. Both cases showed brown regular globules, structureless brown and blue pigmentation, glomerular vessels, hypopigmented regression-like areas, and keratosis. These findings were similar to the cases reported previously. The dermoscopic diagnosis of pBD is based on the absence of criteria for a melanocytic lesion in the presence of glomerular vessels, regular brown globules and keratosis. Although pBD is rare, it should be included in the differential diagnosis of PLs, especially melanoma.

## 1. Introduction

Pigmented lesions (PLs) often have similar characteristics that overlap and can mislead a clinician's diagnosis. Dermoscopy has been shown to increase the diagnostic accuracy of doubtful melanocytic lesions and is often used in the differential diagnosis of PLs like melanoma and pigmented basal cell carcinoma (pBCC) [1, 2]. Bowen's disease (BD), or squamous cell carcinoma *in situ,* is a malignant epithelial tumor that rarely, in patients with dark skin types, may manifest as a pigmented Bowen's disease (pBD) [3, 4]. Dermoscopic features of pBD have been reported previously, but in contrast to other PLs, the dermoscopy of pBD is not specific, and the diagnosis is often a difficult one.

## 2. Objective

To show the dermoscopic features of 2 cases of pBD and compare with other findings reported in the literature.

## 3. Materials and Methods

Two equivocal dermoscopic images of biopsy proven pBD were retrospectively analyzed for the presence of dermoscopic patterns. Both cases were seen in the surgical department of the General Hospital “Dr. Manuel Gea González” in June and September 2009. Demographic data such as age, sex, and Fitzpatrick's skin type were noted. Tumor localization, clinical appearance, and clinical diagnosis were obtained in both cases. 

A DermLite ProHR (3Gen, LLC, San Juan Capistrano, CA, USA) dermoscope mounted on a Canon Rebel camera was used for 10-fold magnification dermoscopic images. The images were taken and analyzed by an experienced dermatologist. The analysis was based on the absence of criteria for melanocytic lesion according to pattern analysis and the two step method [5], and the presence of global and local dermoscopic features that have been previously reported to be suggestive of pBD [1, 2, 4, 6–10] such as: (A) structureless homogenous blue, white, or brown pigmentation, (B) gray or brown regular pigment dots and globules, (C) linear arrangement of pigment dots or globules, (D) peripheral radial streaks (E) vascular pattern: (i) dotted vessels, (ii) glomerular vessels, (iii) linear or clustered arrangement of glomerular vessels, (F) keratosis, (G) ulceration, and (H) additional findings not reported previously. 

Biopsy specimens were stained with hematoxylin and eosin (H&E) and Fontana Masson (FM) and analyzed by two experts in both cases.

## 4. Results

**Case 1**

**Figure 1:**
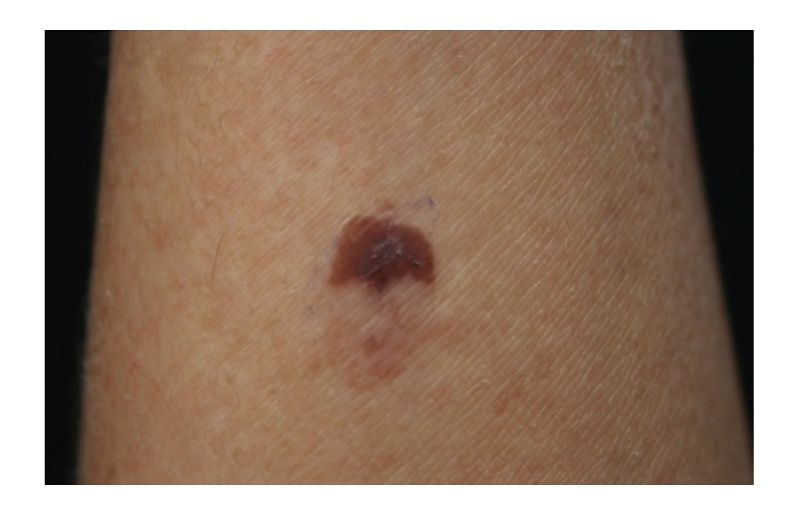
Case 1. Pigmented lesion on the anterior aspect of the forearm.

A 43-year-old woman with type IV skin type presented with a 0.8 × 1.6 cm pigmented lesion on the anterior aspect of the forearm (Figure 1). Examination revealed a flat oval shaped plaque with a sharp border. The upper third of the lesion showed a slightly elevated, sharply demarcated, concave brown discoloration with central keratosis, and a lighter, patchy hue of brown on the inferior two thirds. She had the lesion for two years, but consulted because of enlargement and pruritus during the last four months. She had inactive systemic lupus erythematosus managed intermittently with oral steroids for 1 year, without current treatment.

**Figure 2:**
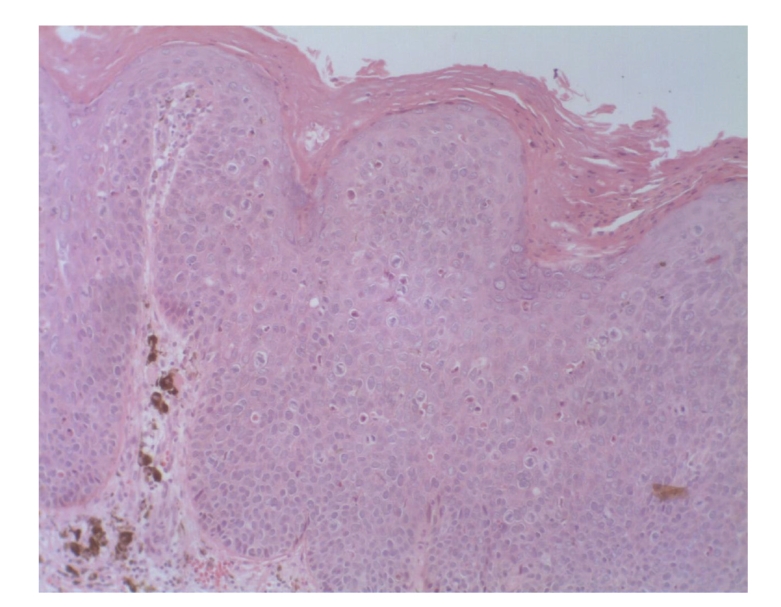
Case 1. H&E shows pBD with parakeratosis and marked acanthosis consisting in atypical keratinocytes on all levels of the epidermis, dilated capillary vessels, and grouped melanophages only in the papillary dermis.

At that time, we considered that dermoscopy showed no criteria for a melanocytic lesion, seborrheic keratosis (SK), or basal cell carcinoma (BCC). Based on the two-step method of pattern analysis of a nonmelanocytic lesion with irregular dotted vessels and pigment remnants, the diagnosis of melanoma was considered. Excisional biopsy was done under the clinical impression of a SK, melanocytic nevus, or melanoma. Biopsy revealed pBD. H&E (Figure 2) showed parakeratosis and marked acanthosis consisting of a disarray of atypical keratinocytes on all levels of the epidermis. The basal layer had abundance of dendritic melanocytes. There were dilated capillary vessels and grouped melanophages only in the papillary dermis. FM highlighted melanin pigment in corneocytes, dendritic melanocytes, dermal melanophages, but only scarcely in keratinocytes.

A complete excision with 5 mm margin was performed and the 8-month followup was uneventful.

**Case 2**

**Figure 3:**
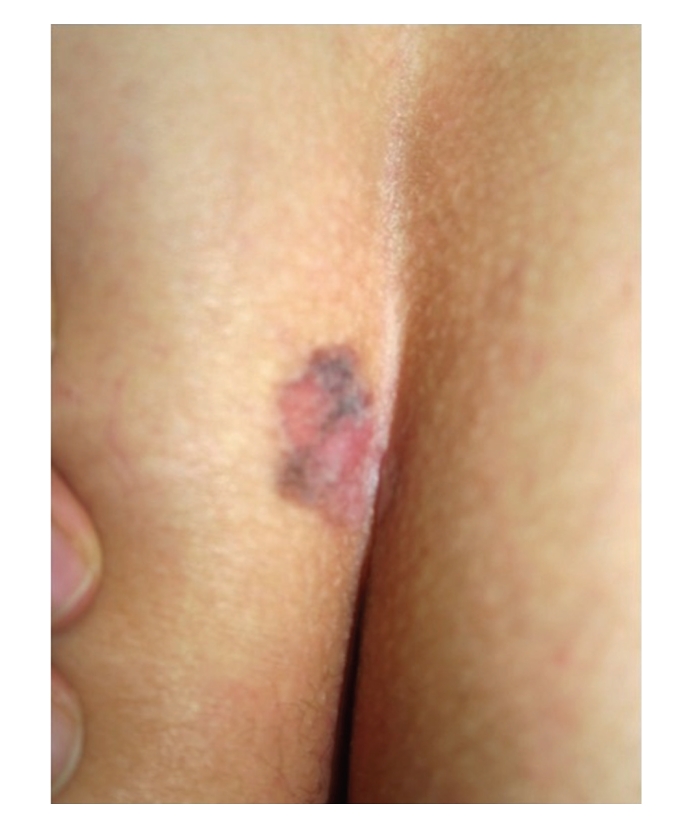
Case 2. Pigmented lesion on the left buttock and intergluteal fold.

A 52-year-old woman with type III skin type consulted for a long-standing pigmented lesion on the left buttock and intergluteal fold (Figure 3). Examination revealed a single 1.2 × 1.6 cm irregular, scaly plaque. It had well-defined borders and heterogeneous discoloration. The center of the lesion had a pinkish white scar-like area and a peripheral poorly defined brown and blue discoloration. The surface was elevated, with verrucous and vegetating areas. It had been noticed 5 years before during a routine gynecological revision, but since the lesion was asymptomatic, she failed to consult a dermatologist until she presented to our clinic for enlargement of the lesion. The patient was otherwise healthy.

**Figure 4:**
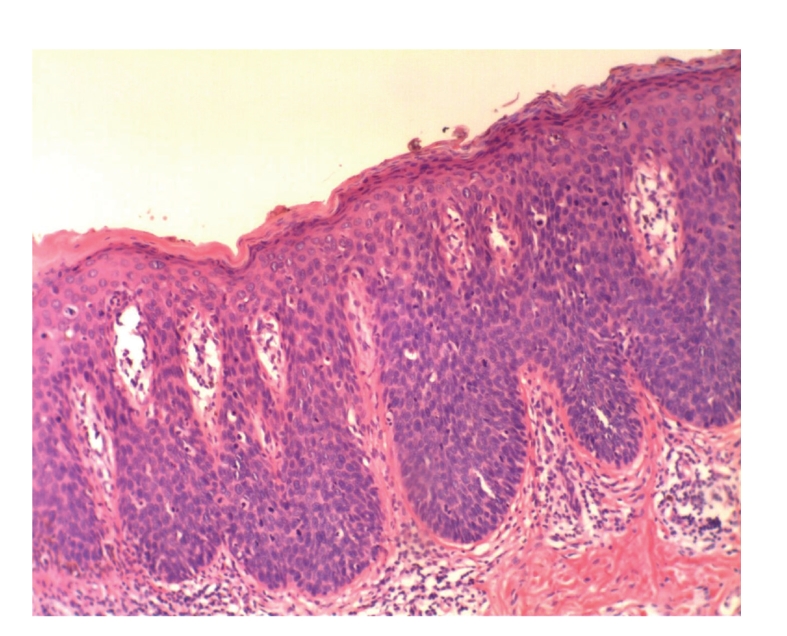
Case 2. H&E shows pBD with marked hyperkeratosis, parakeratosis and acanthosis consisting of atypical keratinocytes on all levels of the epidermis. Dilated capillary vessels and grouped melanophages can be seen in the papillary dermis.

At that time, we considered that dermoscopy showed no criteria for a melanocytic lesion, SK, or BCC. Based on the two-step method of pattern analysis of a non-melanocytic lesion with irregular dotted vessels and pigment remnants, the diagnosis of melanoma was considered. Incisional 4 mm punch biopsy was done under the clinical impression of a melanoma or BD. Biopsy revealed pBD. H&E (Figure 4) showed marked hyperkeratosis and parakeratosis. Acanthosis consisting in a disarray of atypical keratinocytes affected all levels of the epidermis. There were dilated capillary vessels and grouped melanophages in the papillary dermis and none at lower levels. FM highlighted melanin pigment in dendritic melanocytes and dermal melanophages, but not in keratinocytes.

A complete excision with 5 mm margin was performed and the 11-month followup was uneventful.

**Figure 5:**
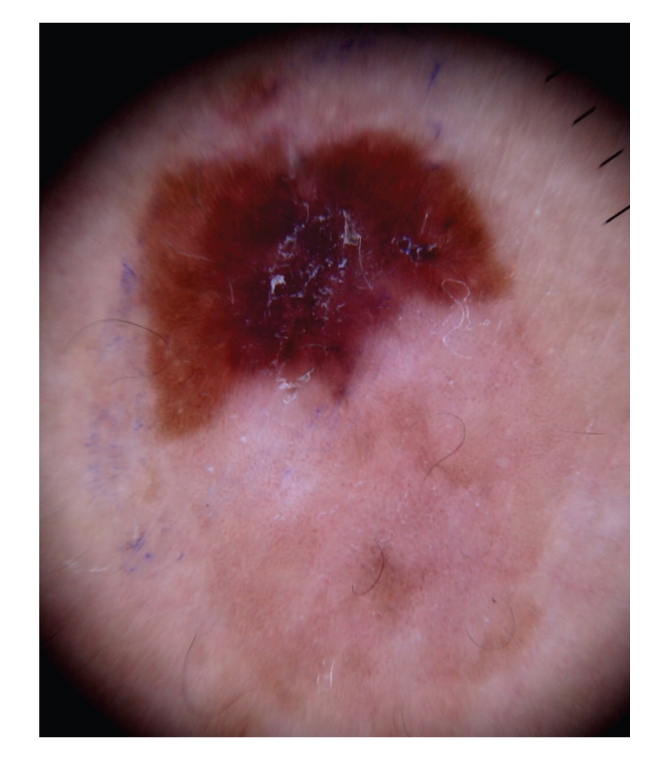
Dermoscopy in Case 1 shows central structureless brown and blue-gray pigmentation with keratosis, irregular peripheral pigmented streaks and brown regular globules in a linear arrangement. On the lower two thirds, light brown regular globules in linear and clustered arrangement, patches of clustered glomerular vessels, and a structureless hypopigmented central area.

**Figure 6:**
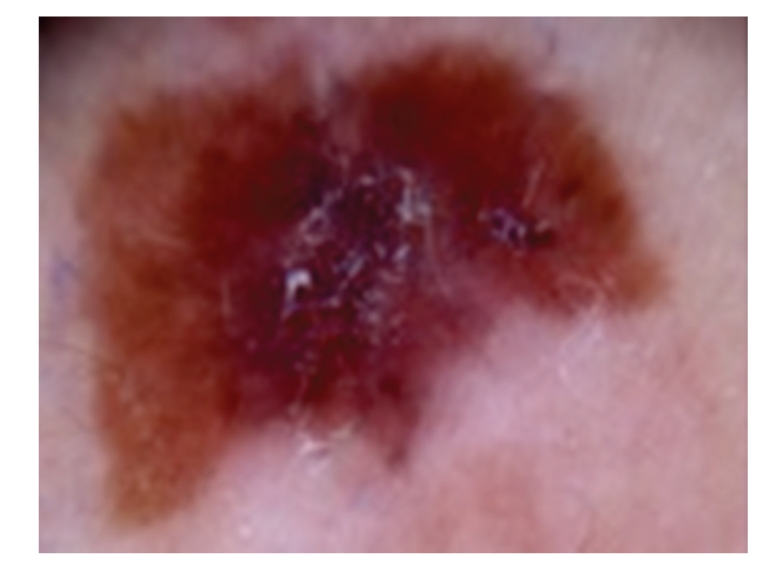
Case 1. Closeup view of upper third of the lesion with peripheral streaks, central brown and blue-gray pigmentation with keratosis and brown globules in a linear arrangement.

**Figure 7:**
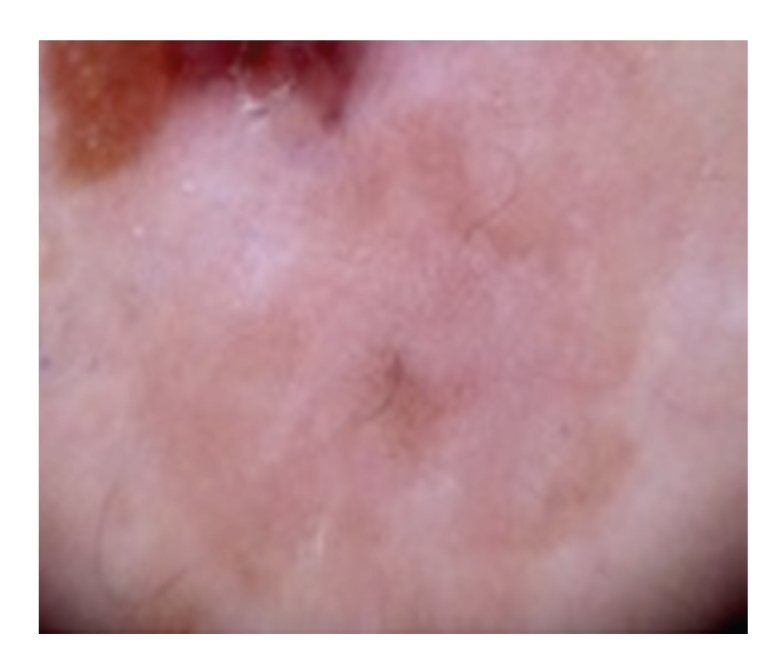
Case 1. Closeup view of lower third of the lesion with patches of pigment globules and glomerular vessels in clustered and linear arrangement.

Retrospective analysis of dermoscopic images of Case 1 revealed, on the upper third of the lesion, absence of specific criteria for a melanocytic lesion, central structureless brown and blue-gray pigmentation with keratosis, irregular peripheral pigmented streaks, and brown regular globules in a linear arrangement. On the lower two thirds, there were light brown regular globules in linear and clustered arrangement, patches of clustered glomerular vessels, and a structureless hypopigmented central area (Figures 5, 6 and 7).

**Figure 8:**
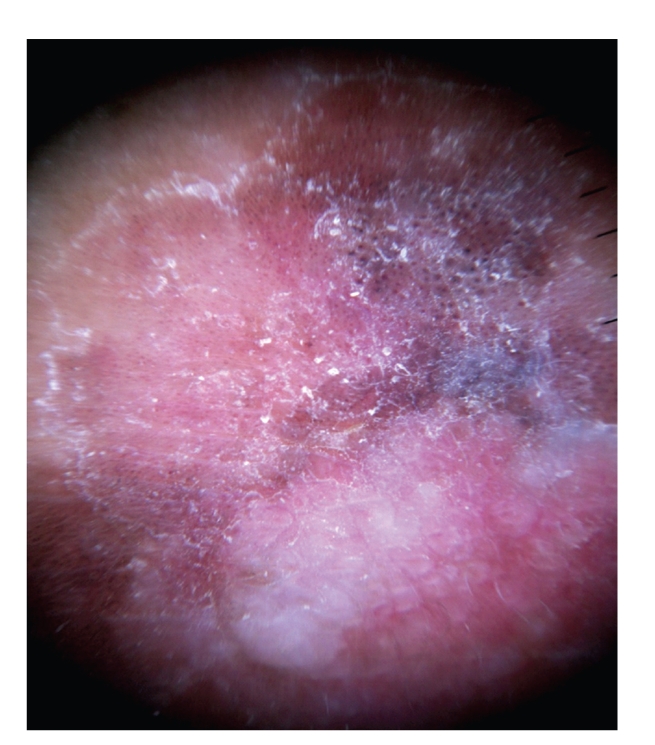
Dermoscopy in Case 2 shows structureless brown and blue-gray peripheral pigmentation with keratosis, patches of brown regular globules in clustered a linear arrangement, clusters of glomerular vessels in a linear arrangement, keratin globules (“cotton candy” keratosis) scattered throughout the lesion, as well as a linear keratinous rim.

**Figure 9:**
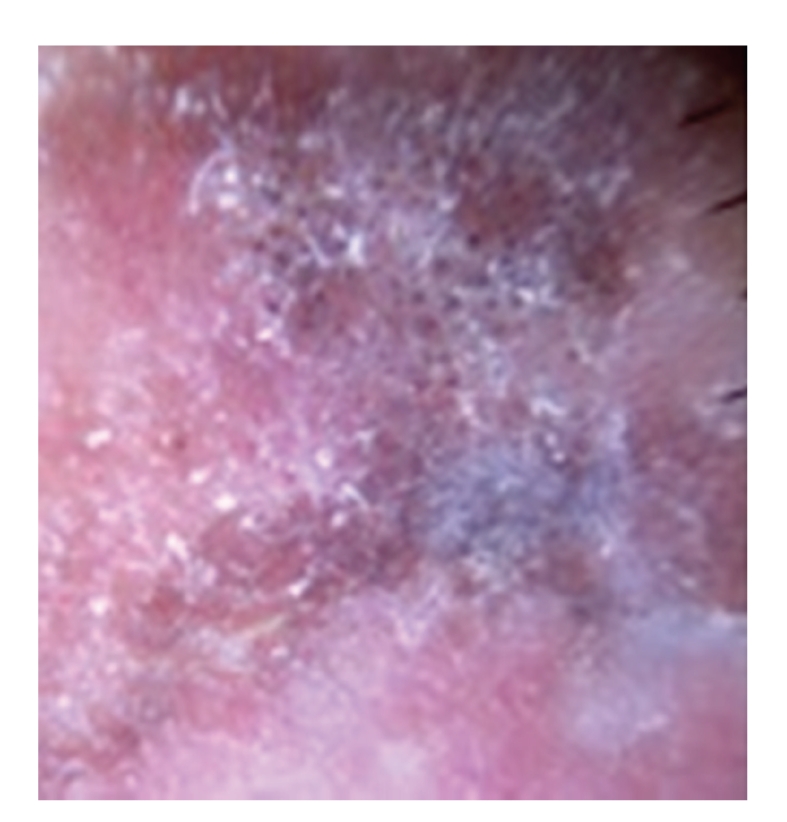
Case 2. Closeup view of clusters of regular pigment globules and glomerular vessels in a linear arrangement. Central area shows brown and blue-gray structureless areas and keratosis.

**Figure 10:**
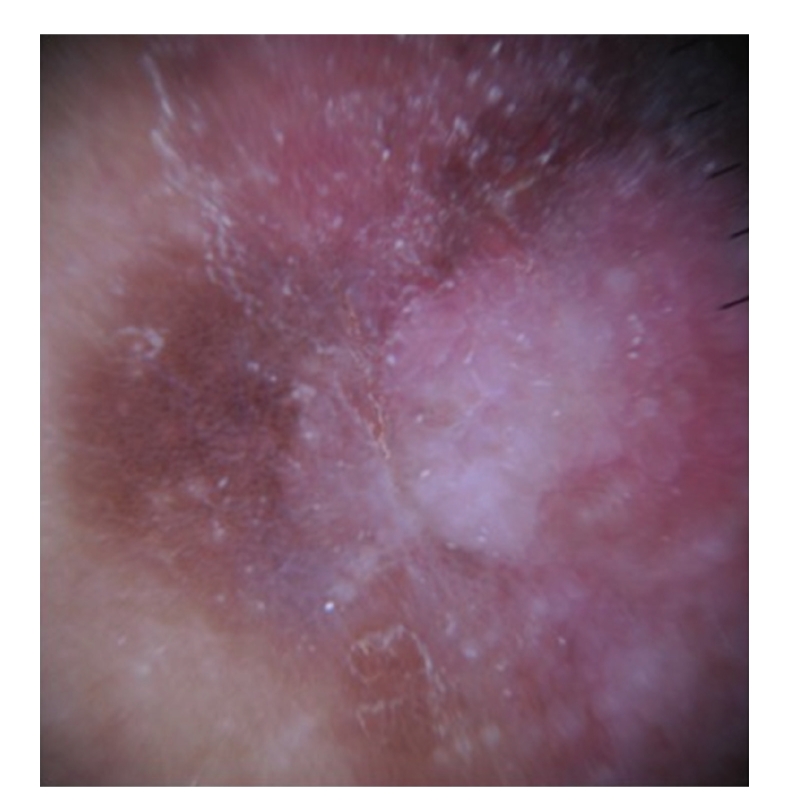
Case 2. Closeup view of “cotton candy” keratosis, keratinous rim and peripheral brown globules in linear arrangement.

Retrospective analysis of dermoscopic images of Case 2 revealed structureless brown and blue-gray peripheral pigmentation with keratosis, patches of brown regular globules in clustered a linear arrangement (Figures 8 and 9), patches of glomerular vessels in clusters an linear arrangement, keratin globules (cloud-like or “cotton candy” keratosis), scattered throughout the lesion (Figure 10), as well as a linear keratinous rim.

## 5. Discussion

BD is a malignant intraepithelial tumor that affects older adults, especially women. Typically, it presents as a slowly enlarging, flat, pink, scaly plaque on sun-exposed areas of Caucasian individuals [4]. Exposure to ultraviolet radiation is the dominant causative factor, but chemicals (arsenic), immunosuppression, and infection with human papillomavirus have also been implicated. Histopathologically, it is characterized by atypical keratinocytes that involve the full thickness of the epidermis [10].

The pigmented variant of BD is rare, and represents only 1.7% [11] to 6% [10] of all cases. Traditionally pBD has been described as more frequently seen in dark skinned individuals with involvement of sun-protected areas, lower extremities, almost exclusively on intertriginous areas [3].

The exact mechanism of the pigment deposition in pBD is not known, although it may arise in association to a SK in up to 13.6% of cases [10, 12]. It has been postulated that it may correspond to the collision with a solar lentigo [10] or to the pigment normally present in the anogenital area [3], but it is also true that many BD may induce pigmentation in the absence of these scenarios [10].

Recently, in the largest publication of pBD, Cameron et al. reported that the most common clinical presentation was a flat or slightly elevated, sharply demarcated, light brown, or variegated papule or plaque with varying degrees of scaling that occurred in men (60%) with an average 67 years of age, on the extremities (44%), followed by the trunk (39%), and head or neck (17%) [10]. Ragi et al. also reported a predominance in men, with an average 61 years of age, none of the lesions presented on the genital area, and interestingly all patients were white [11].

Our cases were 2 women in their mid adulthood, with dark skin type. Just like in Case 1, most of the lesions have been reported in the extremities whereas the appearance of pBD in genital areas, like Case 2 is rare, and there are only 4 reports of other pBD of the genital area [3, 8, 13, 14]. Both were flat, pigmented, and irregular scaly plaques. There were no proven predisposing factors in our cases; Case 1 had a history of intermittent immunosuppressant, nevertheless it is not enough to be considered a causative factor. Case 2 was not tested for the presence of HPV and therefore cannot be ruled out as a predisposing factor. 

PBD should be differentiated from other PLs like SK, pigmented actinic keratosis, solar lentigo, pBCC, melanocytic nevus, blue nevus, melanoma, keratoacanthoma, angioma, and angiokeratoma [1, 2, 15]. These PLs may have similar clinical features that overlap and complicate the differential diagnosis; in fact, the overall sensitivity in diagnostic accuracy between PLs is only 50%. A study showed that only half of the diagnoses of PLs by expert dermatologists were correct [16].

Dermoscopy allows the visualization of pigmented structures in the epidermis and upper dermis [2]. It is used in the differential diagnosis of melanocytic tumors and other PLs, and increases the diagnostic sensitivity to 95% [17, 18]. Nevertheless, differentiating between pBD and other PLs by dermoscopy has been proven difficult. The diagnosis of pBD was not successfully reached by clinical-dermoscopical correlation in previous reports. In these cases, pBD was most frequently confused with melanoma, followed by other PLs like SK, pBCC, and blue nevus [1, 2, 4, 7–9]. 

Dermoscopic findings of pBD were first described in 2004 in two different case reports by Zalaudek et al. [1] and Stante et al. [4]. Zalaudek described a case of pBD with diffuse homogenous blue pigmentation, irregularly distributed, blue-gray granular structures, ulceration, and scaly whitish areas surrounding the ulceration. Stante reported a case of pBD with reticular arrangement of melanin pigment resembling remnants of atypical pigment network, irregular brown peripheral globules and wide regression-like areas. In this paper, two expert dermoscopists could not reach a consensus on the diagnosis of the lesion. 

That same year, in another report, Zalaudek et al. [2] described 10 cases of pBD showing glomerular vessels (80%), scaly surface (90%) small brown globules regularly packed in a patchy distribution (90%), and a grey homogenous pigmentation (80%) as well as pigment network and streaks. Similar findings were later corroborated by other authors [6–10] (see Table 1 [1, 2, 4, 6–10]). 

In a study of 951 cases of BD, Cameron et al. [10] reported the dermoscopic features of 52 cases of pBD. 48% of pBD had structureless pattern only, 35% showed a combination of structureless and dotted pattern, and 17% showed other patterns. With regard to pigmentation, 71% were only brown, 27% were brown and gray, and 2% were only gray. In addition to pigmented areas, hypopigmented (pink, skin-colored, or white) structureless zones were present in 67%. If dots were present, they were either brown or gray, or brown and gray. In all cases of pBD, the distribution of pigment resulted in a variegated appearance. An important dermoscopic clue in pBD was the appearance of brown or gray dots arranged in a linear fashion in 21% that occurred most often at the periphery of the lesion with the lines oriented radially. In 67%, vessels were detected by dermoscopy. The majority (82.9%) showed predominance of one type of vessel; there were coiled vessels (44%), dotted vessels (15%), and other types of vessels (10%). Coiled or dotted vessels were arranged of in a linear fashion in 12% of cases, the coils were elliptical, with the long axis of the ellipses oriented in the direction of the line. Clustered vessels were found in only 6%. There was no clue to the diagnosis in 10%. 

The first report by Zalaudek et al. [1], considered a blue homogeneous pattern together with keratosis a clue to the diagnosis of pBD. The same author [2] in a more detailed report of 10 cases confirmed a combination of glomerular vessels and scaly surface plus homogenous blue pigmentation or pigment globules, as the most common pattern encountered in pBD. Cameron et al. [10], after studying 53 patients with pBD, concluded that the structureless brown pattern was the most frequent. As ‘‘structureless” is the most frequent, but least specific dermoscopic pattern, it is also poses the biggest diagnostic challenge, especially for its implication in the differential diagnosis with melanoma. 

PLs can present as homogenous pigmentation and lack other signs making differentiation difficult. Homogenous blue areas are can also be encountered in blue nevus, pBCC and melanoma. Blue nevus has no other criteria but a homogeneous blue coloration, while BCC has other characteristics like arborizing vessels and maple leaf areas. Coiled vessels can help in the distinction from melanoma and allow the diagnosis of pBD. On the other hand, a pBD with a structureless pattern and no vessels may not be able to be specifically diagnosed by dermoscopy alone. Histopathologically, the homogenous blue pigmentation seen by dermoscopy corresponds to the presence of large amounts of melanin, melanophages, or tumor cells within the papillary dermis [1]. Blue-gray structureless areas resembled regression like areas of a melanoma in Case 2, but lacked other criteria for melanoma like irregular vessels, and pigment network.

**Figure 11:**
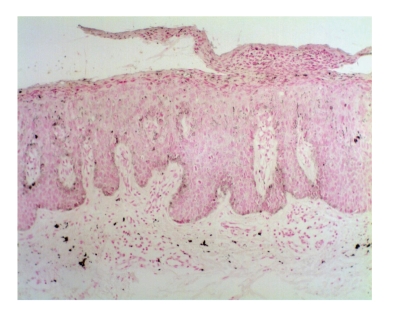
Case 1. Fontana Masson stain highlights pigment in corneocytes, dendritic melanocytes, dermal melanophages, and scarcely in keratinocytes.

**Figure 12:**
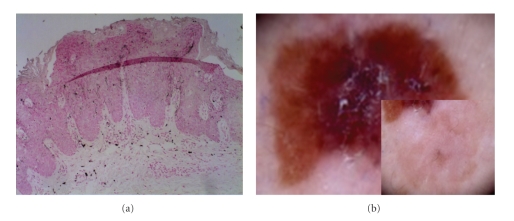
Case 1. Fontana Masson highlights pigment in dendritic melanocytes (a) correlating with pigmented peripheral streaks (b). The scarce pigment in keratinocytes correlates with a structureless brown pattern and the melanophages with the blue-gray areas and clusters of brown globules (bottom b).

**Figure 13:**
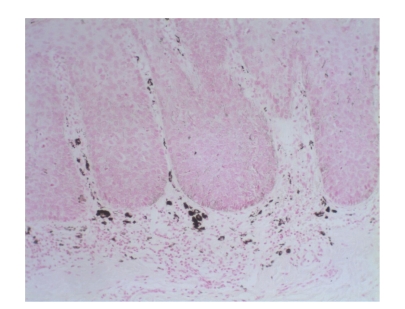
Case 2. Fontana Masson stain highlights melanin pigment in dendritic melanocytes and dermal melanophages, but not in keratinocytes.

Brown pigment corresponds to melanin in keratinocytes. FM in Case 1 showed pigmentation in corneocytes, dendritic melanocytes, and superficial dermal melanophages, and only scarcely in keratinocytes (Figures 11 and 12). In our case, pigment correlated with the presence of melanophages and dendritic melanocytes since keratinocytes were not heavily pigmented. In Case 2, FM showed melanin pigment in dendritic melanocytes and dermal melanophages but none in keratinocytes (Figure 13). If only basal keratinocytes are pigmented, the pattern observed is structureless brown. Because of the marked acanthosis of the epidermis with loss of rete ridges, hyperpigmentation of basal keratinocytes does not usually result in reticular lines (‘‘pigment network”), and can be encountered in only (4%). Hypopigmentation correlates with absence of pigmented basal keratinocytes [10].

**Figure 14:**
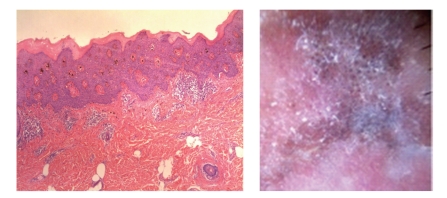
Case 2. H&E. Melanophages arranged in groups in the papillary dermis that correlate with brown globules and dilated capillary vessels correlate with glomerular vessels.

The second most common pattern encountered by Cameron et al. was the combination of dots and structureless pattern (hypopigmented being the most common). In practice, the most frequent differential diagnosis of this pattern will be SK and various benign and malignant melanocytic lesions and, less commonly, pBCC. Case 2 (Figure 14), revealed structureless brown and blue-gray peripheral pigmentation with keratosis, patches of brown regular globules in clustered a linear arrangement and patches of glomerular vessels in clusters and linear arrangement, all conclusive of pBD.

Pigment globules can also be seen in melanoma, blue nevus and pBCC, and make the diagnosis of a melanocytic lesion difficult, especially if the lesion lacks other diagnostic criteria for pBCC or melanocytic lesion [1]. But pigment globules in pBD have been shown to be smaller, arranged regularly, in clusters [2, 7] or lines [10]; pigment globules represent melanophages arranged in clusters in the superficial dermis, or highly pigmented keratinocytes in the basal layer. In melanocytic lesions, globules correspond to nests of melanocytes in the dermoepidermal junction, but also to melanophages in the superficial dermis [2]. Small collections of pigment in higher levels of the epidermis correspond to brown dots. Gray dots, on the other hand, correspond to melanophages in the papillary dermis. The linear arrangement of dots and vessels observed in some cases of pBD could not clearly be clearly explained [10]. FM is positive in both melanocytes and keratinocytes without evidence of a significant change of melanocyte distribution or increase in number with Melan-A [2]. These were clues for the diagnosis of pBD in both of our cases, where regular small brown globules in a patchy and linear distribution were seen. In both cases, melanophages arranged in groups in the papillary dermis correlated with brown globules (Figure 14).

A pigmented network correlates with melanocytes in the basal layer and is the most specific criteria for melanocytic lesion [4]. Although we did not encounter pseudonetwork, or reticular pigmentation like Stante et al. [4] Hu et al. [7], and Bugatti et al. [6], Case 1 had pigmented streaks simulating pigmented remnants that prompted us to rule out a melanocytic lesion. Pigment network was not found in past publications of pBD except in the report by Cameron et al. [10], which encountered it in 4% (see Table 1). These “false” melanocytic parameters are not uncommon in non-melanocytic lesions; 10% of pigmented SK shows melanocytic criteria [19]. There are dermoscopic differences between reticular pigmentation and pigment network, considering that the brownish lines in reticular pigmentation correspond histologically to the melanin pigment in the dermal papillae whereas the lines in the pigment network, a true melanocytic clue, correspond histologically to the pigmented melanocytes in rete ridges [7].

**Figure 15:**
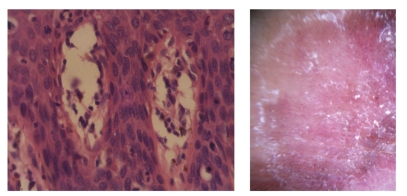
Case 2. H&E. Closeup of dilated capillary vessels in the papillary dermis enclosed by the marked acanthosis that correlates with glomerular vessels.

Vascular patterns are distinctive for certain tumors and can be used to discriminate between different PLs. Glomerular vessels are specific for pBD as seen in both of our cases; 100% of BD and 80% of pBD presented glomerular vessels with an irregular and patchy pattern [2]. Glomerular vessels represent the convolution of grouped dilated vessels in the dermal papillae and papillary dermis [2, 10]. Case 2 showed dilated capillary vessels in the papillary dermis enclosed by the marked acanthosis that correlates with the presence of clusters of glomerular vessels (Figures 14 and 15).

Glomerular vessels are larger than dotted vessels, regular, and arranged in clusters. The linear arrangement of glomerular is a specific clue to pBD [10]. Regular dotted vessels can also be found in psoriasis, viral warts, and clear cell acanthoma, but psoriasis and warts are easy to differentiate by clinical exam, and acanthoma has a linear or string of “pearls” pattern to the dotted vessels. Amelanotic melanoma can also have dotted vessels, but linear arrangement of vessels and keratin can help distinguish between pBD and melanoma [2]. 

**Figure 16:**
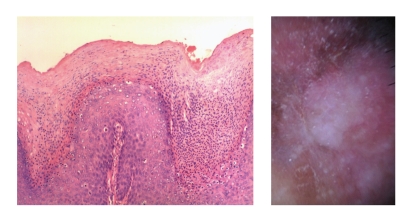
Case 2. Marked hyperkeratosis and parakeratosis correlates with “cotton candy” pattern keratosis.

PBD is a keratinous tumor, and therefore scaly surface has been reported as a dermoscopic hallmark in 90% of cases of BD, and the majority of pBD where it can be a diagnostic clue to distinguish between pBD and other PLs like pBCC and melanoma [1, 2, 6–8]. Scaly surface correspond to a hyperkeratotic and parakeratotic stratum corneum [6]. Both cases presented keratosis. In Case 1, it was present as a ring-like peripheral border of the lesion. Case 2 had keratin globules in the center of the lesion, where marked hyperkeratosis and parakeratosis correlated with “cotton candy” pattern (Figure 16). These patterns of keratosis had not been described previously. 

Since none of our cases fulfilled criteria for a melanocytic lesion by dermoscopy, the diagnosis was based on pathological correlation. These findings are similar to the cases reported in the literature, where none were diagnosed correctly by clinical or dermoscopic exam [1, 2, 4, 7–9]. A clinical-dermoscopical-pathological correlation was made in retrospect in our study. Nevertheless, a multicomponent pattern, clustered small pigment globules especially at the periphery of the lesion in a linear arrangement, and clustered glomerular vessels in a linear pattern, in the absence of other criteria for a melanocytic lesion or pBCC can be used in the diagnosis of PBD. Keratosis can also be an important clue. These 2 cases, and the other 81 reported in the literature [1, 2, 4, 6–10] exemplify that pBD can sometimes lack a specific pattern, or the pattern may be similar to other PLs; therefore, pBD should be included in the differential diagnosis of all PLs.

## 6. Conclusion

PBD shares similar features with other PLs including pBCC and melanoma. Although dermoscopy increases the diagnostic sensibility of PLs, diagnosis of pBD continues to be a challenge. Dermoscopic diagnosis of pBD should be should be considered in the differential diagnosis of PLs when a pigmented tumor with absence or doubtful criteria of a melanocytic lesion is associated with blue structureless areas, dotted vessels and scaly surface, or in the presence of specific findings of regular clusters of glomerular vessels and brown globules, especially in a linear arrangement. Although pBD is rare, it should be included in the differential diagnosis of PLs, especially melanoma.

## Figures and Tables

**Table 1:** Dermoscopic features of pigmented Bowen's disease from various reports.

Author	Patients	Findings
Zalaudek et al. [1]	1	Homogeneous blue pigmentation
		Irregularly distributed, blue-gray granular structures
		Black to dark brown crust
		Scaly whitish areas

Stante et al. [4]	1	Reticular pigmentation resembling remnants of atypical
		pigment network
		Irregular brown globules at the periphery
		Regression-like areas

Zalaudek et al. [2]	10	Glomerular vessels 80%
		Scaly surface 90%
		Regular pigment globules in a patchy distribution 90%
		Grey-brown homogenous pigmentation 80%

Bugatti et al. [6]	14	Multicomponent pattern (100%)
		Atypical vascular structures (87%)
		(Dotted, linear, arborizing, bushy, and hairpin-like vessels)
		Pseudonetwork (36%)
		Irregular diffuse pigmentation or blotches of pigment (64%)
		Irregularly distributed dots and globules (64%)
		Focal/multifocal hypopigmentation (79%)
		Scaly surface (64%)
		Hemorrhages (27%)

Hu et al. [7]	1	Glomerular vessels
		Scaly surface
		Small brown globules regularly packed in a patchy distribution
		Reticular pigmentation
		Grey-brown homogeneous pigmentation

Hernández-Gil et al. [8]	1	Irregular pigment globules
		Atypical vascular pattern (“rounded vessels”)
		Scaly surface

De Giorgi et al. [9]	1	Radial streaks regularly distributed in the periphery
		Irregular hypopigmented veiled scar-like regression area
		Brownish and reddish globules

Cameron et al. [10]	52	One pattern present (54%)
		Two patterns present (46%)
		Symmetrical arrangement (12%)
		Asymmetrical arrangement (88%)
		Only structureless pattern (48%)
		Pattern of dots and/or structureless zones (35%)
		Hypopigmented structureless zones (67%)*
		Brown or gray dots arranged in a linear fashion (21%)*
		Vessels (67%)
		Coiled vessels (44%)*
		Dots (15%)
		Linear arrangement of coiled vessels (12%)*
		Clustered vessels (6%)*
		Pigment network (4%)

*Clues to the diagnosis.
